# Potential role of chimeric genes in pathway-related gene co-expression modules

**DOI:** 10.1186/s12957-021-02248-9

**Published:** 2021-05-12

**Authors:** Piaopiao Li, Yingxia Li, Lei Ma

**Affiliations:** College of Life Science, Shihezi University, Shihezi, Xinjiang, 832000 China

**Keywords:** Chimeric gene, Domain, WGCNA, Co-expression network, Tumor

## Abstract

**Background:**

Gene fusion has epigenetic modification functions. The novel proteins encoded by gene fusion products play a role in cancer development. Therefore, a better understanding of the novel protein products may provide insights into the pathogenesis of tumors. However, the characteristics of chimeric genes are rarely studied. Here, we used weighted co-expression network analysis to investigate the biological roles and underlying mechanisms of chimeric genes.

**Methods:**

Download the pig transcriptome data, we screened chimeric genes and parental genes from 688 sequences and 153 samples, predict their domains, and analyze their associations. We constructed a co-expression network of chimeric genes in pigs and conducted Gene Ontology enrichment and Kyoto Encyclopedia of Genes and Genomes pathway analysis on the generated modules using DAVID to identify key networks and modules related to chimeric genes.

**Results:**

Our findings showed that most of the protein domains of chimeric genes were derived from fused pre-genes. Chimeric genes were enriched in modules involved in the negative regulation of cell proliferation and protein localization to centrosomes. In addition, the chimeric genes were related to the growth factor-β superfamily, which regulates cell growth and differentiation. Furthermore, in helper T cells, chimeric genes regulate the specific recognition of T cell receptors, implying that chimeric genes play a key role in the regulation pathway of T cells. Chimeric genes can produce new domains, and some chimeric genes are a key role involved in pathway-related function.

**Conclusions:**

Most chimeric genes show binding activity. Domains of chimeric genes are derived from several combinations of parent genes. Chimeric genes play a key role in the regulation of several cellular pathways. Our findings may provide new directions to explore the roles of chimeric genes in tumors.

**Supplementary Information:**

The online version contains supplementary material available at 10.1186/s12957-021-02248-9.

## Introduction

Chimeric genes are produced from the fusion of two or more parent genes [1] through chromosomal rearrangements, transcriptional read-through of adjacent genes, trans-splicing, and other mechanisms. Chimeric genes can be translated into new proteins with novel functions. Although proteins encoded by chimeric genes can show beneficial functions, the encoded proteins can also have deleterious functions. Chimeric genes are a cytogenetic feature of many cancers and have been used as diagnostic markers [2]. For example, the *EML4* gene and the *ALK* gene are fused into a chimeric gene [3], which has been used as a marker for advanced non-small cell lung cancer. Investigation into the protein domains encoded by chimera may help provide insights into the cellular functions of the encoded proteins.

The features of the proteins produced by chimeric genes depend on the domains produced by parental genes. For example, in chronic myelogenous leukemia, the high tyrosine kinase activity of the chimeric BCR-ABL protein is derived from the fusion of the phosphorylation domain encoded by the *BCR* gene with the non-receptor tyrosine kinase domain encoded from the *ABL* proto-oncogene [4]. In early prostate cancer, the expression of erythroblast virus E26 carcinogen gene 2 (ERG) is increased through its fusion with the trans-membrane serine protease two gene (TMPRSS2) [5]. However, the principle that chimeric genes inherit domains from their parents requires further study. Normally, signal peptides (SP) direct chimeric proteins to their proper cellular and extracellular locations [6]. They are involved in the discovery of drug targets, protein production, and cancer biomarkers [7]. For example, the macrocyclic triamine cyclotriazadisulfonamide (CADA) decrease expression of specific proteins in a SP-dependent manner has opened the door to the possibility that the signal peptide becomes a validated target for drug design [8]. The signal peptide missense variant in cancer-brake gene CTLA4 was associated with lower risk and poor prognosis in breast carcinoma among Egyptian women, might have prognostic as well diagnostic impact in breast cancer [9]. Therefore, we took signal peptides as an example to explore the source of chimeric gene protein domains.

Gene design is a strategy to manufacture protein-encoding genes with specific biological functions. In these methods, gene sequences that encode different protein domains are fused to produce a fusion protein product with specific functions. For example, the artificially synthesized MGF-Ct24E peptide induces migration-promoting activity in human myogenic precursor cells and may be helpful for the treatment of Duchenne muscular dystrophy [10]. However, not all artificial fusion proteins perform the desired functions. For example, synthetic oligopeptides with selectin agglutination domains reduced ischemic damage at 24 h after transient focal cerebral ischemia, but did not reduce permanent focal cerebral ischemia [11]. Therefore, a better understanding of the characteristics of endogenous chimeric genes may be useful to guide gene design and synthesis.

Pigs have been used as large mammal models in various research studies [12]. Pigs are highly similar to humans not only in body weight, physiological characteristics, organ formation, and disease occurrence, but also in genomic sequence and chromosomal structure [13]. To explore the structural characteristics of chimeric genes in pigs, we used weighted co-expression network analysis (WGCNA) to investigate the role of chimeric genes in the network. Our results showed that the formation of a chimeric gene not only enrich the diversity and complexity of the transcription and protein, and provides a reference for the study of human chimeric genes.

## Materials and methods

### Data preparation

To define chimeric mRNA sets, mRNA datasets were downloaded from the Nucleotide database of the National Center for Biotechnology Information (NCBI, http://www.ncbi.nlm.nih.gov/nucleotide/, September 2016) [14], containing a total of 688 sequences (see Additional file 1). Pig reference genome sequence (Sus Sscrofa 10.2) was downloaded from the Ensembl Genome Browser (http://asia.ensembl.org/index.html, September 2016) [15]. Then, the mRNA reads were aligned to a pig reference genome sequence (Sus Sscrofa10.2). When a single mRNA sequence was aligned to multiple locations of the reference genome, only 0.5% of the homology level of the reference genome sequence was retained and at least 96% of the gene sequences were identical to the mRNA sequence. We obtained 1007 chimeric RNAs.

### Prediction of chimeric and parental protein domains

We download the ncbi-blast-2.2.25 -x64-Win64 version to build a local BLAST (ftp://ftp.ncbi.nlm.nih.gov/blast/executables/blast+/LATEST) [16] and compared 1007 chimeric mRNA with the mRNA dataset to predict the parent genes of the chimeric genes. Parameters were set as follows: (i) similarity (% identity) > 95%, (ii) left and right base alignment lengths > 90%, and (iii) *E* value of the comparison < 10^−5^ [17]. A total of 447 chimeric mRNAs matched to two parent genes.

We used Open Reading Frame Finder (ORF Finder, http://www.bioinformatics.org/sms2/orf_find.html) [18] from NCBI to predict the amino acid sequence of the chimeric mRNAs. The parameter settings were as follows: (i) minimal ORF length: 75 nt, (ii) ORF start codon: “ATG” only, (iii) genetic code: standard, (iv) amino acid length: greater than 100, and (v) the positive chain: retained.

SMART (http://smart.embl-heidelberg.de/) [19] and Universal Protein Resource (UniProt, http://www.uniprot.org/uniprot/?query=pyrin&sort=score) [20] were used to predict the domains encoded by chimeric and parental genes. The parameter setting was as follows: remove the hidden| overlap domain. SignalP4.1 (http://www.cbs.dtu.dk/services/SignalP/) was used to predict sequences encoding signal peptides in chimeric and parent genes [21].

### Enrichment analysis of chimeric and non-chimeric genes

The functional enrichment analysis of chimeric and nonchimeric genes was performed using the Database for Annotation, Visualization and Integrated Discovery (DAVID, https://david.ncifcrf.gov/) [22], and the false discovery rate (FDR) value less than 0.05 indicated significance. The pig genome level was used as the background for statistical analysis of enrichment.

### Construction of the gene co-expression network

Download the pig transcript expression, including 153 samples (see Additional file 2): (i) if the number of genes expressing 0 in a sample accounts for more than 20% of the total number, the sample is deleted; (ii) genes with expression standard deviation greater than 5 were selected; and (iii) cluster samples and delete outliers.

We used the WGCNA package [23], dynamicTreeCut package [24], and FastCluster package [25] in R (version 4.02) to construct a co-expression network for pigs. The specific process is described in reference [26].

### Functional enrichment of module

Functional enrichment analysis was performed using DAVID [27], using an FDR value of less than 0.05 to indicate significance. The pig genome level was used as the background for statistical analysis of enrichment.

### Pathway involved in chimeric genes

We used DAVID for Kyoto Encyclopedia of Genes and Genomes (KEGG) path mining [27], using an FDR value less than 0.05 to indicate significance. The background of statistical analysis is based on the genome level of pigs.

## Results

### Distribution of chimeric domains

Domains of 1007 chimeric genes were predicted, and 1942 protein domains and 582 protein domain types were obtained. We analyzed the distribution frequency of the 582 protein domain types (excluding Signal peptides) and found that most domains only appeared once or twice. The results showed that only the 3% (20/582) of chimeric domain occurrences number greater than 15, such as ZnF, coiled coil, WD40, EFh, LIM, HAT, and IG.

In order to obtain the domain indicators that can be used as fusion events, we compared the top 20 chimeric domains with the porcine genome domains and found that WD40, EFh, RRM, SH3, and SH2 were significantly enriched in the chimeric domains (Fisher’s exact test, *p* < 0.001, Table 1). In addition, the overall distribution rate of chimeric protein domains is similar to the distribution of pig genome protein domains (Fisher’s exact test, *p* = 0.3582, Table 1).

**Table 1:** Distribution characteristics of chimeric domains

Data set	Overall gene number	Gene number (domains)	Domain type	Overall domain number	WD40 (%)	EFh (%)	RRM (%)	SH3 (%)	LRR (%)	SH2 (%)	ANK (%)
Chimeric	1007	868	582	1942	8.9	5.3	4.0	3.3	2.9	2.7	2.7
Genome	25882	23300	5943	59,701	2.9	0.7	0.7	0.5	2.2	0.3	2.3
*P*			> 0.05		< 0.01						

Note: *P*, the result of Fisher’s exact test

### Signal peptides encoded by the chimeric

To provide a real-world example on the origin of domains encoded by chimeric mRNAs, we used the signal peptide as an example. A signal peptide is a 5–30 amino acid peptide located in the N-terminus of secretory proteins. Signal sequences have a tripartite structure, consisting of a hydrophobic core region (h-region) flanked by an n-region and c-region. The latter region contains the signal peptidase consensus cleavage site.

**Fig. 1:**
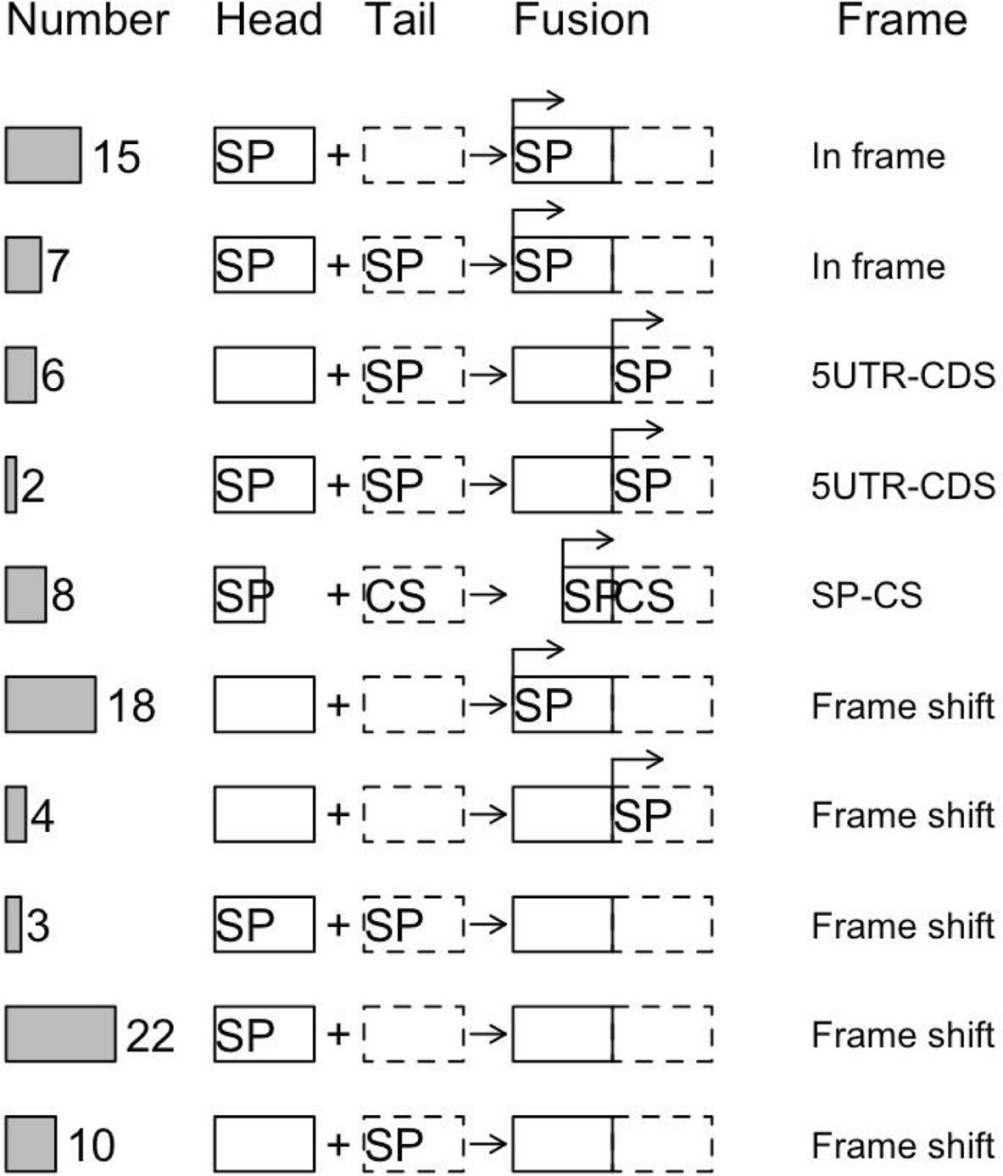
Gain and loss of signal peptide in fusion genes. SP, signal peptide; CS, cleavage site. UTR, untranslated region; CDS, coding sequences. The rectangular arrow on the fusion gene box indicates the starting position of the translation

As shown in Fig. 1, the chimera can obtain signal peptides through several mechanisms. (i) The signal peptides can be derived from the head parent (HP), (ii) the signal peptide can be derived from the tail parent (TP), regardless of whether the HP has the signal peptide; the HP became untranslated region (UTR) and the TP offered coding sequences, forming a 5′ UTR-coding sequence structure, and (iii) signal peptides can be re-built by connecting parent sequences. For example, an incomplete signal sequence of the HP obtained a cleavage site from the TP and (iv) reading-frame shift either creates or destroys signal peptide.

### Comparison of protein domains between chimeric and parental genes

Among the 1007 chimeric genes, there are 447 two parent chimeric genes, 430 one parent chimeric genes, and 130 chimeric genes with no results. Analysis of each of 447 chimeric genes that matched two parent genes showed that although these chimeric genes contained domains of the parent genes, the chimeric genes were not just a combination of their two parent protein domains. Approximately 61% (273/447) of these chimeric genes retained the domains of their parent genes. Among the 273 chimeric genes, 52 were identical with their parental genes, 94 retained the domain of one parent gene, and 127 contained domains of the two parent genes. The remaining 106 chimeric genes (24%, 106/447) contained novel domains not found in parent genes. Approximately 15% (68/447) of the chimeric gene does not contain the domain of their parent.

There were 338 domain types in the 447 chimeric genes, and their sources were analyzed statistically (Table 2). A total of 140 domain types were derived from only one parent gene. Among the 140 domain types, 60 types come from 5′ parent genes, 80 types come from 3′ parent genes, 78 types come from both parent genes, 34 types resulted in a reading frame shift, and 86 types have no confirmed source.

**Table 2:** Source of domains in chimeric genes

Number of chimeric domains	5′ parent	3′ parent	Source
60	+	−	5′ parent
80	−	+	3′ parent
78	+	+	5′ parent and 3′ parent
34	−	−	Reading frame shift
86	+/−	+/−	5′ parent or 3′ parent or reading frame shift

“+”: the domain of the chimeric gene is derived from the parent gene. “−”: the domain of the chimeric gene does not belong to the parent gene. “/”: or

### Construction of chimeric gene co-expression modules

**Fig. 2:**
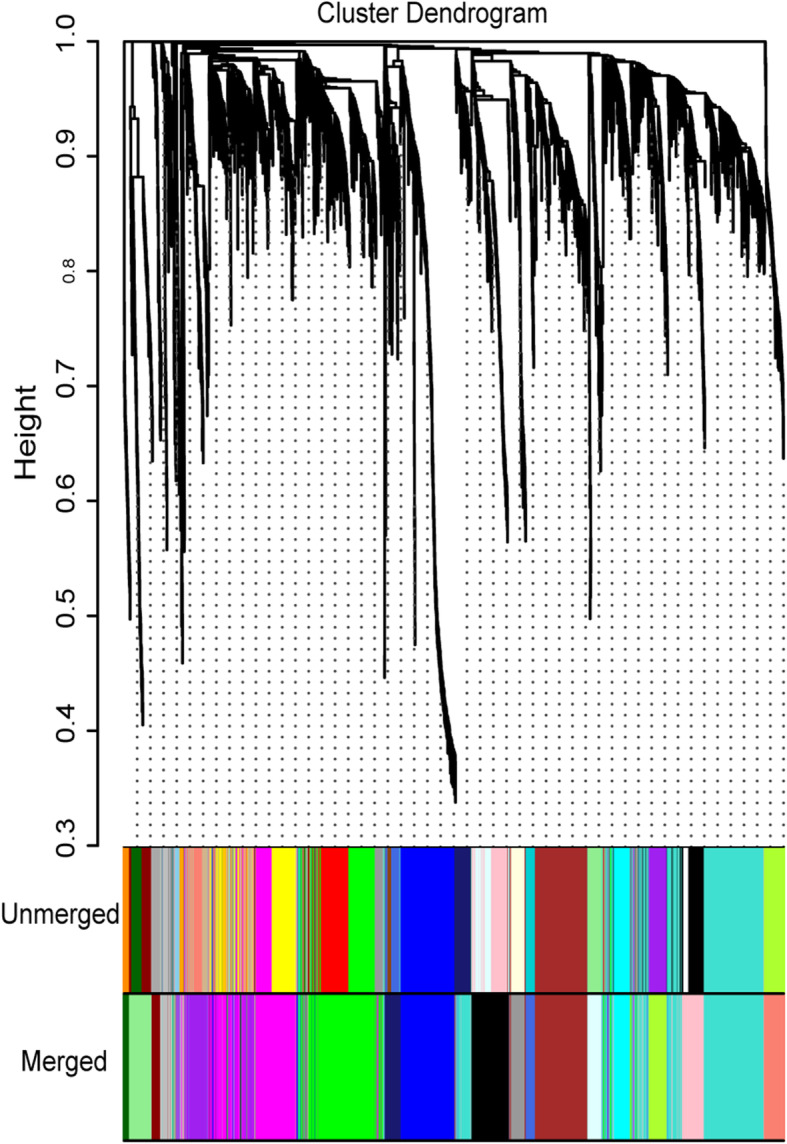
The cluster of transcriptions and construction of modules. Different colors represent different modules. Cluster dendrogram, transcriptions cluster; unmerged, preliminary module construction; merged, integrated module

Using the abundance values of 475 chimeric genes and 2433 non-chimeric genes in 153 pig RNA-sequencing samples, we constructed 19 gene co-expression modules (Fig. 2; Table 3). The number of transcripts varied in the modules. The largest module, #1, contained 479 transcripts while the smallest module #19 contained only 32 transcripts. Furthermore, the number of chimeric transcripts also varied in the modules.

**Table 3:** Co-expression network module information containing chimeric genes

#	Color in Fig. 2	Cluster gene number	Number of chimeric genes	Chimeric proportion
1	Turquoise	479	80	16.70%
2	Green	367	60	16.30%
3	Magenta	324	55	17.00%
4	Blue	262	51	19.50%
5	Brown	250	35	14.00%
6	Purple	185	29	15.70%
7	Black	178	27	15.20%
8	Pink	126	13	10.30%
9	Lightgreen	103	14	13.60%
10	Greenyellow	83	13	15.70%
11	Salmon	83	17	20.50%
12	Cyan	82	11	13.40%
13	Midnightblue	72	13	18.10%
14	Grey	67	14	20.90%
15	Grey60	62	10	16.10%
16	Lightcyan	61	12	19.70%
17	Royalblue	49	5	10.20%
18	Darkred	43	8	18.60%
19	Darkgreen	32	8	25.00%

Cluster gene number indicates the total gene number in each module, the number of chimeric genes indicates the chimeric gene number in each module, and the chimeric proportion indicates the number proportion chimeric genes in each module

### Functional enrichment analysis

**Fig. 3:**
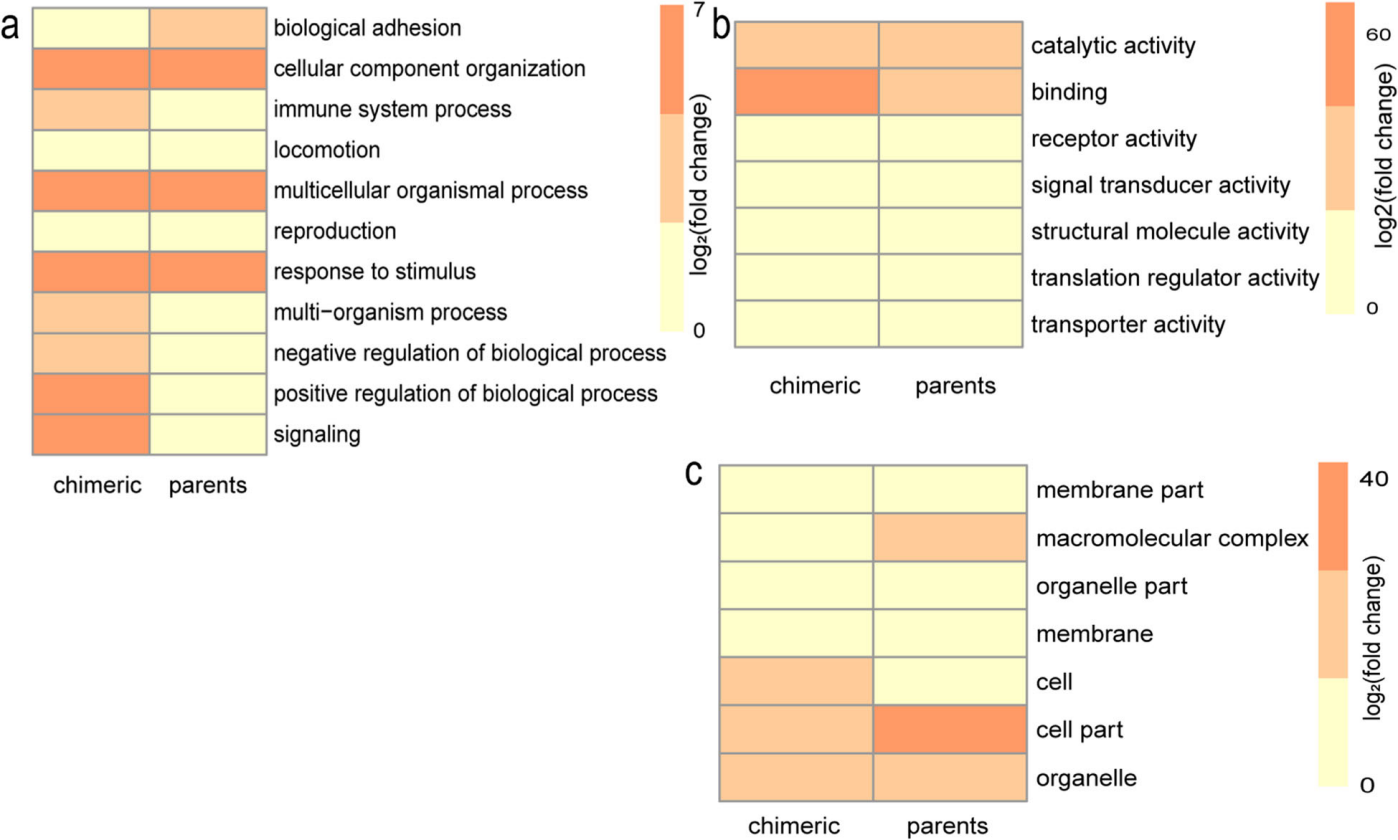
The number distribution of chimeric genes and parental genes in **a** biological processes, **b** molecular functions, and **c** cell component. The ordinate indicates function. The abscissa indicates chimeric and parental genes.

Enrichment analysis using DAVID showed that the functions of chimeric genes were different compared with those of non-chimeric genes (FDR < 0.05). For biological processes, chimeric genes were enriched in biologic regulation and single organism process while non-chimeric genes were enriched in cellular and metabolic processes (Fig. 3a). For molecular functions, chimeric genes showed functions in binding while non-chimeric genes showed functions in catalytic activity (Fig. 3b). For the cytology component, chimeric genes are involved in cells while non-chimeric genes are involved in organelles (Fig. 3c).

### Functional enrichment analysis in specific modules

**Fig. 4:**
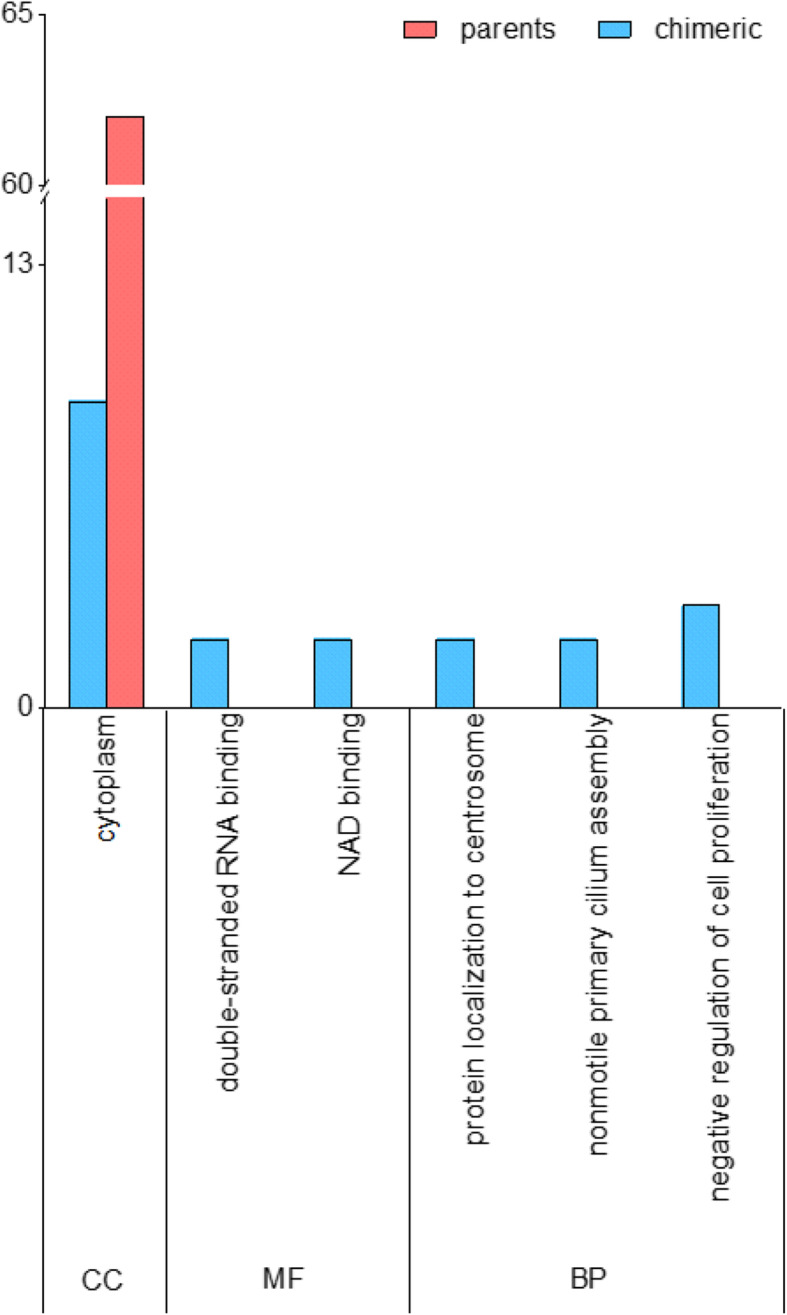
The distribution of chimeric gene function enrichment in turquoise module. The abscissa indicates function. The ordinate indicates genes number. BP, biological processes; CC, cell component; MF, molecular function

The functional correlation of genes between modules was validated by enrichment analysis of the chimeric genes in modules #1–5. The chimeric genes in different modules were enriched to the same function. Module #1, module #3, and module #4 were enriched in the cytoplasm while module #5 was enriched in the nucleus. Module #2 was enriched in extracellular exosomes. However, in module #1, the chimeric genes were enriched in different functions (Fig. 4).

### Module visualization

**Fig. 5:**
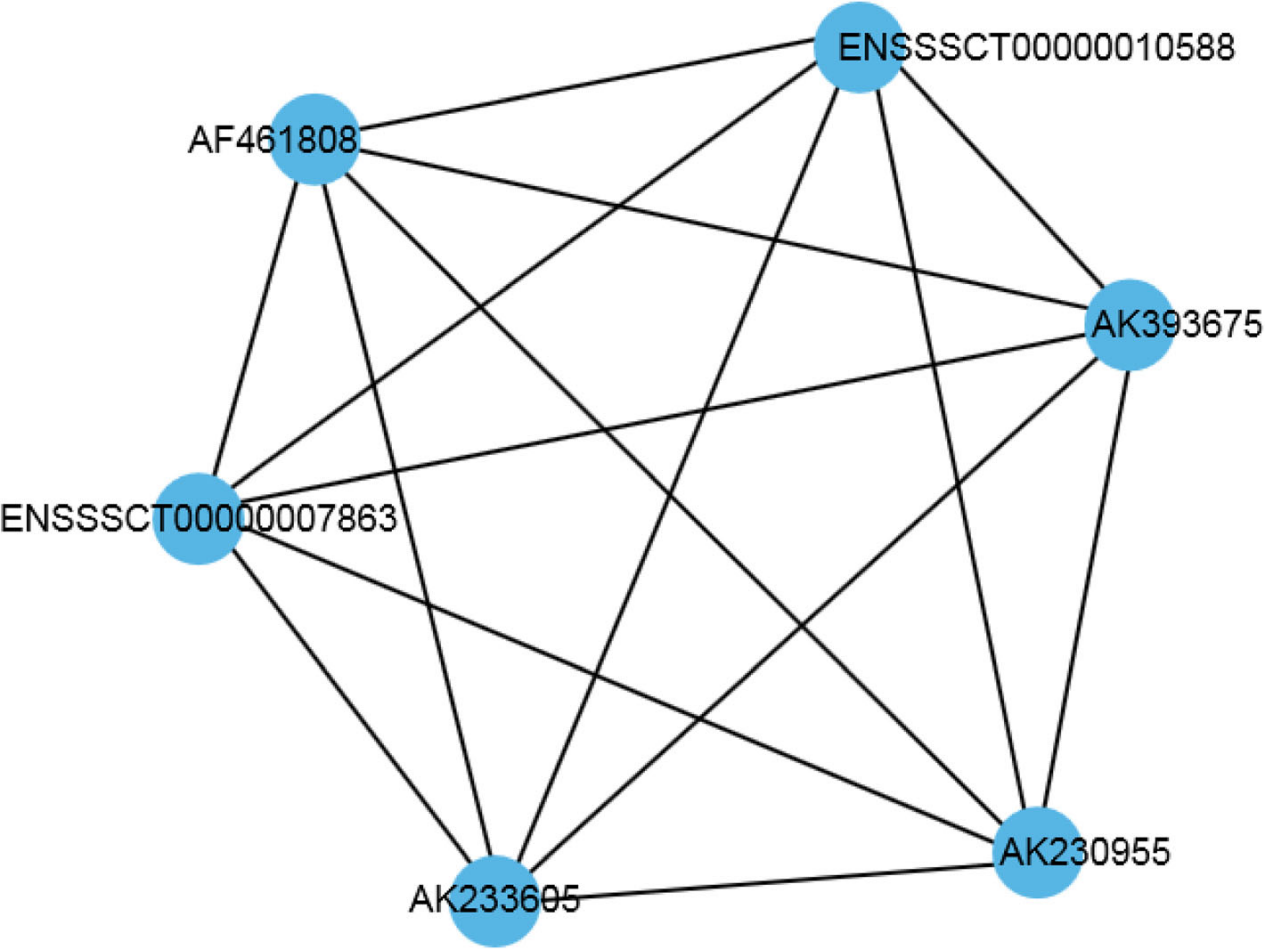
The gene co-expression regulatory network of the third module and the fourth module. Line, a correlation between genes. The blue circle, gene registration number

The relationship between chimeric and non-chimeric genes in the network was revealed by analyzing the co-expression of genes in modules #4 and module #5. As shown in Fig. 5, the chimeric genes appear more frequently than non-chimeric genes. This network is mainly related to the transforming growth factor-β superfamily, which plays a role in regulating cell growth and differentiation. In this network, chimeric genes (AK461808, AK393675, AK233605, AK230955) and non-chimeric genes (ENSSSCT00000010588, ENSSSCT00000007863) are connected to each other. They can regulate each other.

### Chimeric genes are involved in the regulation of T cells

**Fig. 6:**
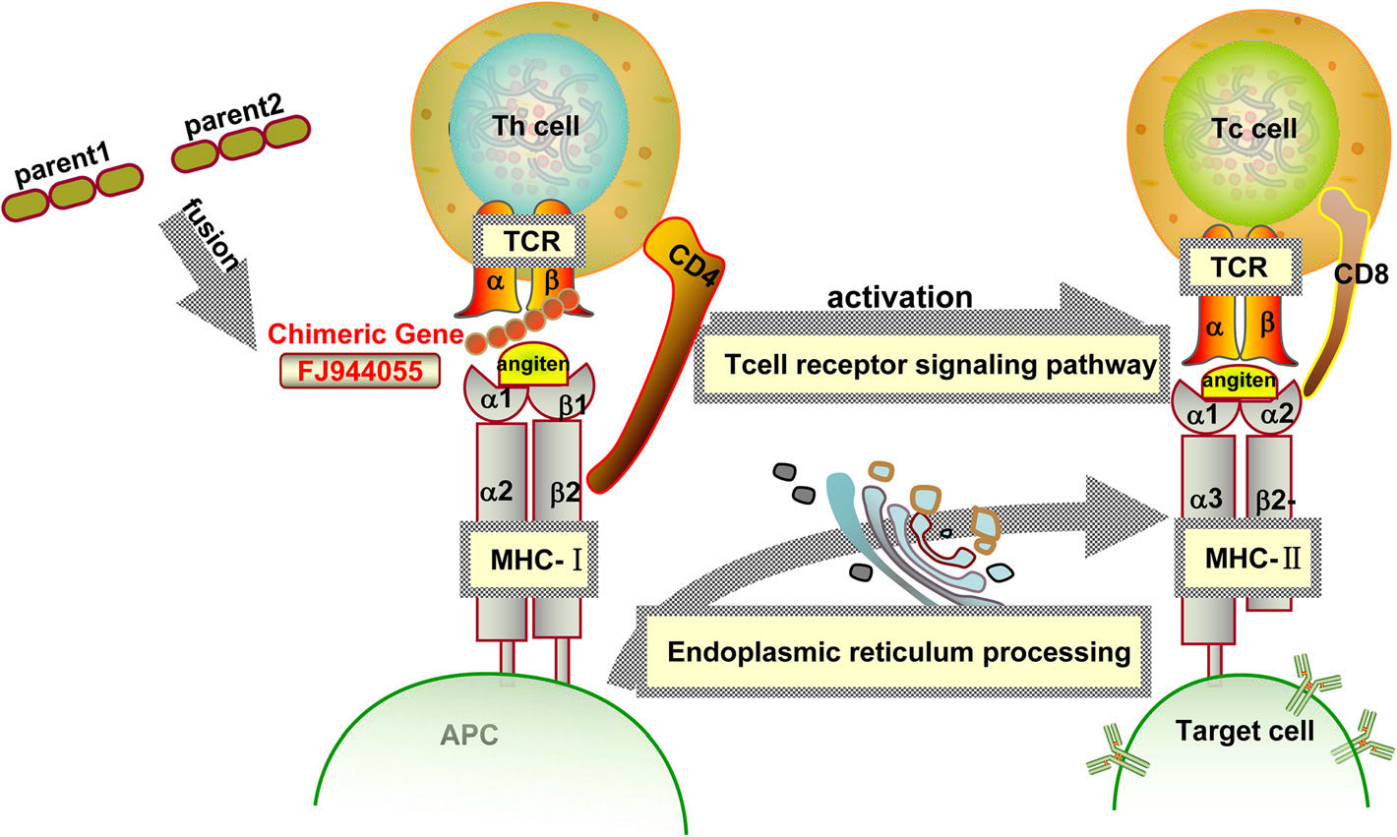
The regulatory pathway of T lymphocytes. Th cell, helper T cell; Tc cell, cytotoxic T cell, TCR, T cell antigen receptor; APC, antigen-presenting cells

We identified relationships between chimeric and non-chimeric mRNAs in various cellular pathways. As shown in Fig. 6, the chimeric gene (FJ944055) encodes the T cell antigen receptor (TCR) beta chain, which forms the TCR cell with the alpha chain. The chimera (FJ944055) can regulate the TCR to identify the antigen presented by the MHC molecule. Non-chimeric genes (AB602431, AK397194) are involved in the regulation of MHC class I (MHC-I) and MHC class II (MHC-II) molecules. MHC-I and MHC-II molecules bind to T cell antigen receptor (TCR) to activate CD8+ T cells and CD4+ T cells, respectively.

## Discussion

The domains encoding by chimeric genes can be derived from parental genes in various ways. The domains and functions of a chimeric gene may be the same as those in parent genes. For example, when genes that encode oncoproteins are fused, the chimeric genes may encode proteins that accelerate the division of cancer cells. However, most chimera encode both parental and novel domains. In cases in which a chimeric gene has a new function compared with the parent gene, it will suppress or promote the expression of the parent [28].

We used the signal peptide as an example to provide a real-world example on the origin of domains encoded by chimeric genes. A signal peptide is composed of about 5–30 amino acids and guides the transport of proteins through the cell membrane [6]. Signal peptides play different roles in chimeric genes. The LPCAT2-TXNDC5 chimeric product is derived from fusion of the *LPCAT2* gene, which contains a signal peptide–encoding sequence, with the *TXNDC5* gene, which lacks this sequence [29]. LPCAT2-TXNDC5 chimera is detected extracellular space, possibly from the protein being transported through the membrane. The gene that does not encode protein products can fuse with other genes, resulting in a fusion gene with protein-encoding capability. This may be due to signal peptides provided by other genes or proteins produced by a reading frameshift.

WGCNA can be used to find modules of highly related transcripts, help screen hub transcripts and identify candidate biomarkers [30]. Using WGCNA, we found that genes in the same module are functionally related to each other [31]. Chimeric genes and parent genes in the same module can simultaneously edit hexokinase. This result is consistent with the study showing that the *MYB-QKI* chimeric gene regulates the same pathway as the parental gene [28]. The results also revealed specific regulatory relationships between chimeric genes and non-parent genes in different modules. KEGG pathway enrichment analysis revealed that the TCR-beta gene and pig MHC-I and MHC-II transcripts were enriched in viral myocarditis pathways. TCR identifies heterologous antigen through signal regulation, killer T cells identify MHC class I antigen, and helper T cells identify MHC class II antigen. In addition, TCR play functions in cancer, and TCR expression predicts prognosis for non-small cell lung cancer patients after curative surgery [32]. We hypothesized that TCR and MHC antigens recognized by TCR may exist and function in non-small cell lung cancer tissues. More studies are required to explore this possibility. Together these findings suggest regulatory relations between chimeric genes and parental genes in the same module and show that chimeric genes and non-chimeric genes have similar effects in different modules.

The studies on chimeric genes have mainly focused on a specific chimeric gene and explore the relation between its function and cancer occurrence. Our current study provides insights into the general characteristics of chimeric genes and systematically analyzes the role of chimeric genes in co-expression networks. For example, a previous study examined that the *FOXO1-PAX3* chimeric genes as a focus of Alveolar rhabdomyosarcoma (ARMS), exploring its related regulatory network [33]. In the research, we integrated and compared pig transcriptional data with DNA data and identified 1007 chimeric genes. We used these chimeric genes to build a chimeric genes co-expression network using WGCNA. The results revealed a regulatory relationship between chimeric genes and non-chimeric genes. The specific regulatory networks between chimeric genes and non-chimeric genes require further study.

## Conclusions

In conclusion, most chimeric genes show binding activity, and domains of the chimeric genes are derived from several combinations of parent genes. WD40, EFh, RRM, SH3, and SH2 domains may be used as domain indicators for fusion events. In our analyses, we detected differences in the number of chimeric genes in the modules. Chimeric genes play a key role in the regulation of several cellular pathways. These findings may provide new directions to explore the roles of chimeric genes in tumors.

## Supplementary Information

**Additional file 1.** (XLS 80 kb)

**Additional file 2.** (XLS 32 kb)

## Data Availability

All data in this study were obtained from public databases.

## Abbreviations

GO: Gene Ontology
KEGG: Kyoto Encyclopedia of Genes and Genomes
WGCNA: Weighted gene co-expression network analysis
DAVID: Database for Annotation, Visualization and Integrated Discovery
FDR: False discovery rate
HP: Head parent
TP: Tail parent
UTR: Untranslated region
CDS: Coding sequences
CS: Cleavage site
TCR: T cell antigen receptor
